# Navafenterol (AZD8871) in healthy volunteers: safety, tolerability and pharmacokinetics of multiple ascending doses of this novel inhaled, long-acting, dual-pharmacology bronchodilator, in two phase I, randomised, single-blind, placebo-controlled studies

**DOI:** 10.1186/s12931-020-01474-1

**Published:** 2020-09-09

**Authors:** Victor Balaguer, Muna Albayaty, Eulalia Jimenez, Ulrika Wählby-Hamrén, Carol Astbury, Beatriz Seoane, Marie-Pierre Malice, Alejhandra Lei, Ajay Aggarwal, Ioannis Psallidas

**Affiliations:** 1 Research and Early Development, Respiratory, Inflammation and Autoimmune, BioPharmaceuticals R&D, AstraZeneca, Barcelona, Spain; 2 the Early Phase Clinical Unit, PAREXEL International GmbH, Harrow, UK; 3 Clinical Pharmacology and Quantitative Pharmacology, Clinical Pharmacology & Safety Sciences, R&D, AstraZeneca, Barcelona, Spain; 4 Clinical Pharmacology and Quantitative Pharmacology, Clinical Pharmacology & Safety Sciences, R&D, AstraZeneca, Gothenburg, Sweden; 5 Biometrics and Information Sciences, Late Stage Development, BioPharmaceuticals R&D, AstraZeneca, Barcelona, Spain; 6 Early Biostats and Statistical Innovation, Data Science and AI, BioPharmaceuticals R&D, AstraZeneca, Barcelona, Spain; 7 Patient Safety RIA, Chief Medical Office, R&D, AstraZeneca, Barcelona, Spain; 8 Research and Early Development, Respiratory, Inflammation and Autoimmune, BioPharmaceuticals R&D, AstraZeneca, Boston, MA USA; 9 Research and Early Development, Respiratory, Inflammation and Autoimmune, BioPharmaceuticals R&D, AstraZeneca, Cambridge, UK

**Keywords:** Bronchodilator, COPD, MABA, Dual-pharmacology muscarinic receptor antagonist β_2_-adrenoceptor agonist, Safety, Pharmacokinetics

## Abstract

**Background:**

Navafenterol (AZD8871) is a novel, long-acting, dual-pharmacology (muscarinic receptor antagonist and β_2−_adrenoceptor agonist) molecule in development for chronic obstructive pulmonary disease and asthma.

**Methods:**

These two phase I, randomised, single-blind, multiple-ascending-dose studies evaluated inhaled navafenterol and placebo (3:1 ratio) in healthy, male, non-Japanese (study A; NCT02814656) and Japanese (study B; NCT03159442) volunteers. In each study, volunteers were dosed in three cohorts, allowing gradual dose escalation from 300 μg to 600 μg to 900 μg. The primary objective was to investigate the safety and tolerability of navafenterol at steady state. Pharmacokinetics were also assessed.

**Results:**

Twenty-four volunteers completed each study (navafenterol, *n* = 6; placebo, *n* = 2 in each cohort). There were no deaths, serious adverse events (AEs) or treatment-emergent AEs (TEAEs) leading to discontinuation of navafenterol. The most frequent TEAEs were vessel puncture-site bruise (placebo, n = 2; navafenterol 900 μg; *n* = 3) in study A and diarrhoea (placebo, *n* = 1; navafenterol 300 μg, n = 2; navafenterol 900 μg, n = 3) in study B. No dose-response relationship was observed for TEAEs. There was a dose-dependent increase in mean heart rate on day 16 in both studies. The pharmacokinetics of navafenterol were similar between non-Japanese and Japanese volunteers.

**Conclusions:**

Multiple ascending doses of navafenterol were well-tolerated and the safety and pharmacokinetics of navafenterol were similar in non-Japanese and Japanese volunteers. The findings support navafenterol clinical development.

**Trial registration:**

ClinicalTrials.gov; Nos.: NCT02814656 and NCT03159442; URL: www.clinicaltrials.gov.

## Introduction

Chronic obstructive pulmonary disease (COPD) and asthma cause substantial morbidity and mortality worldwide; in 2015, there were 3.2 million deaths from COPD and 0.4 million from asthma [1], and both diseases ranked amongst the top 15 causes of disability [2]. Current treatment for both diseases involves a step-wise approach, and bronchodilator therapy is a key component of this. In COPD, combination treatment with a long-acting muscarinic receptor antagonist (LAMA) and long-acting β_2_-adrenoceptor agonist (LABA) is recommended as a step-up treatment for patients whose COPD is not well managed on initial treatment with LAMA, LABA, or LABA/inhaled corticosteroids (ICSs) [3]. Additionally, combined LAMA/LABA treatment is recommended as initial treatment for patients with a substantial symptom burden [3]. Triple therapy with LAMA/LABA/ICS is an option for patients with persistent symptoms and exacerbation risk [3]. Triple therapy is also used in the management of asthma, with the LAMA tiotropium recommended as add-on therapy for patients aged ≥12 years whose asthma is not well controlled with ICS/LABA therapy [4].

Navafenterol (AZD8871) is a novel chemical entity possessing long-acting, dual-pharmacology (muscarinic receptor antagonist and β_2−_adrenoceptor agonist [MABA]) activities in a single molecule. Preclinical pharmacological characterisations of navafenterol and an earlier compound in the series, LAS190792, have been described previously [5, 6]. The key structural difference between these two compounds is in the region linking the antimuscarinic and β_2_-adrenoceptor functional regions, which is an N-phenylcarbamate in navafenterol and a benzotriazole in LAS190792 [6]. The linker in navafenterol, in contrast to that in LAS190792, tips the balance of activities toward M_3_ antagonism, resulting in a dual bronchodilator with fewer of the secondary effects associated with β-adrenoceptor agonism [6]. The β_2_-adrenoceptor activity of LAS190792 is markedly higher than that of navafenterol and both compounds have low β_1_-adrenoceptor activity [6].

MABAs could represent an alternative therapeutic approach in COPD and, when combined with an ICS, provide simplified formulation development and a single pharmacokinetic (PK) profile compared with LAMA/LABA combinations [7]. Through its dual pharmacological activity, it is anticipated that navafenterol would offer greater efficacy than single-mechanism LAMAs or LABAs, and similar or potentially greater efficacy than free- or fixed-dose combination therapies, with an equivalent or superior safety and tolerability profile. In the first-in-human study, single ascending doses of navafenterol of 50, 200, 400, 900, 1800 and 2100 μg in patients with mild asthma were well tolerated with no safety concerns raised [8]. Clinically meaningful and sustained bronchodilation was observed with doses from 200 to 2100 μg [8]. Navafenterol is intended to be developed for registration worldwide and so it is important to study its effects in different populations since differences in the PK, safety and tolerability, or pharmacodynamic effects of drugs are sometimes observed between individuals of different ethnicities [9]. Here, we present safety, tolerability and PK data for navafenterol after single and repeat dosing in healthy volunteers from different ethnic backgrounds.

## Materials and methods

### Study design

These were two phase I, single-centre, randomised, single-blind, placebo-controlled, multiple–ascending-dose studies of inhaled navafenterol in healthy, male, non-Japanese (study A; NCT02814656) and Japanese (study B; NCT03159442) volunteers. The primary objective of each study was to investigate the safety and tolerability of navafenterol at steady state. The secondary objective was to characterise the PK of navafenterol and its metabolites, LAS191861 and the pharmacologically inactive LAS34850, after single and multiple doses of navafenterol and to assess the time required to reach steady state, the degree of accumulation and the time-dependency. Exploratory pharmacodynamic endpoints (lung function and pupillometry) were also evaluated (e-Appendix 1).

Both studies were conducted at PAREXEL EPCU, Northwick Park Hospital, London, UK. The final protocols and informed consent forms were approved by the local ethics committee (e-Appendix 1). The studies were performed in accordance with the Declaration of Helsinki, the International Council for Harmonisation/Good Clinical Practice guidelines, applicable regulatory requirements and the AstraZeneca policy on Bioethics. Volunteers provided voluntary, written informed consent before taking part in study procedures.

**Fig. 1:**
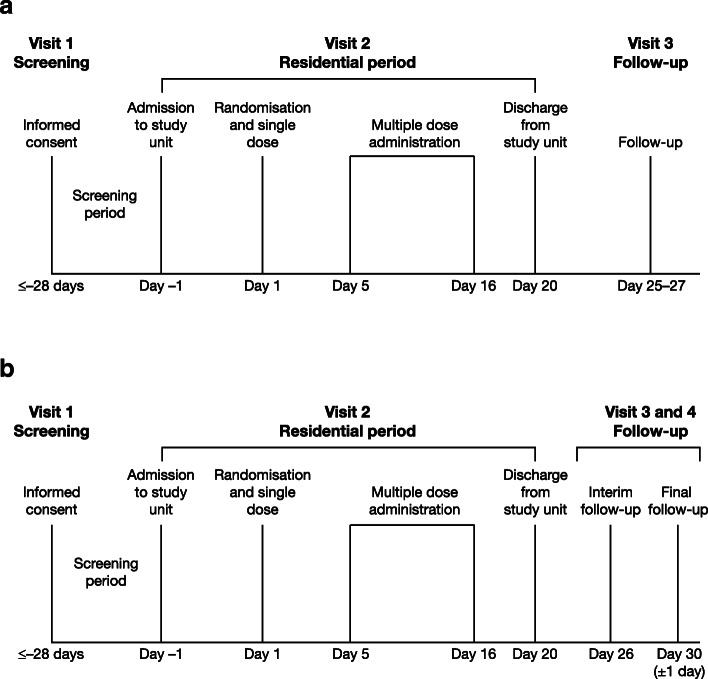
Study design for (**a**) study A and (**b**) study B

Volunteers were admitted the day prior to the first dose (day − 1) and remained on-site until day 20, 96 h following administration of the final dose on day 16 (Fig. 1). In each cohort, volunteers received a single dose of navafenterol or placebo on day 1, followed by a 4-day wash-out period and one dose/day from days 5 to 16. Evaluation and characterisation of PK were conducted after administration of a single dose (PK monitoring for 5 days), then the multiple dose study began with the same subjects so the 20-day residence comprised both single dose and multiple dose studies in the same subjects. Volunteers were randomised 3:1 to receive navafenterol or placebo, both administered via a variant of the Genuair™/Pressair®^1^ dry powder inhaler adapted internally to deliver a single dose of inhalation powder. Devices containing placebo or navafenterol had identical external appearances. There were three cohorts (one for each dose level) and each volunteer only participated in one cohort. The study design allowed a gradual escalation of dose from cohort to cohort with intensive monitoring to ensure the safety of volunteers.

Volunteers in cohort 1 received a single dose of navafenterol 300 μg or placebo. Dose selection for cohorts 2 and 3 was determined by a Safety Review Committee following review of data from the previous cohort. In order to proceed to the next dose level, a minimum of 5 volunteers on active treatment must have completed dosing. The doses selected by the safety review committee for cohorts 2 and 3 were 600 and 900 μg, respectively. Predefined stopping criteria are summarised in e-Appendix 1.

### Volunteers

Males aged 18–55 (study A) or 20–55 (study B) years were eligible for inclusion. In study B, Japanese volunteers were defined as those born in Japan with two Japanese biological parents and four Japanese grandparents and who had not lived outside of Japan for more than 5 years or had a significant change in lifestyle or diet since leaving Japan. Inclusion and exclusion criteria and study restrictions are reported in e-Appendix 1.

### Assessments

#### Safety and tolerability

Safety and tolerability assessments included adverse events (AEs), physical examination, vital signs, clinical laboratory assessments (including serum glucose and potassium i-STAT measurements), 12-lead digital and safety local electrocardiograms (ECGs), and 2-lead real-time telemetry. Assessment timings are outlined in e-Appendix 1.

#### Pharmacokinetics

Blood samples for PK analysis were collected at pre-dose and at 15, 30 and 45 min, and 1, 1.5, 2, 3, 4, 6, 8, 12, 16, 24, 36, 48, 72 and 96 h post-dose on days 1 and 16 and also at pre-dose on days 6 to 15 (both studies) and at the follow-up visits (days 26 and 30, study B only). The plasma concentrations of both navafenterol and its metabolites were assessed using validated bioanalytical assays with a lower limit of quantification of 2 pg/mL (navafenterol and LAS191861) or 25 pg/mL (LAS34850). The PK parameters assessed included: observed maximum concentration (C_max_); time to reach C_max_ (t_max_); terminal half-life (t_½λz_); area under the concentration-time curve (AUC) from time zero extrapolated to infinity (AUC_0-∞_), AUC from time zero to the time of last quantifiable concentration (AUC_last_), and AUC from time zero to 24 h post-dose (AUC_0–24_); accumulation ratio for C_max_ (Rac [C_max_], estimated as the ratio of C_max_ on day 16 to that on day 1); accumulation ratio for AUC_0–24_ (Rac [AUC_0–24_], estimated as the ratio of AUC_0–24_ to that on day 1); and the metabolite to parent ratio for C_max_ (MRC_max_) and AUC_0–24_ (MRAUC_0–24_).

### Statistical analysis

Due to the exploratory nature of the studies, the sample size was not based on formal statistical considerations. It was planned to randomise 24 volunteers in each study (8 volunteers per cohort, 6 receiving navafenterol and 2 receiving placebo). No formal statistical hypothesis testing or corrections for multiplicity were performed.

Demographic and baseline data were summarised by treatment (navafenterol dose), but placebo data from the three cohorts were pooled. Safety data were analysed descriptively for the safety population (all volunteers who received at least 1 dose of investigational drug [navafenterol or placebo] and for whom any post-dose safety data were available).

PK data were analysed in the PK population (all volunteers in the safety population who received at least 1 dose of navafenterol, had at least one of the parameters C_max_, AUC or AUC_last_ evaluable for navafenterol, and were not affected by factors such as protocol deviations). Dose proportionality, time dependency and accumulation were assessed.

Details of the statistical analysis of the PK and pharmacodynamics are provided in e-Appendix 1.

## Results

### Volunteer demographics and baseline characteristics

**Fig. 2:**
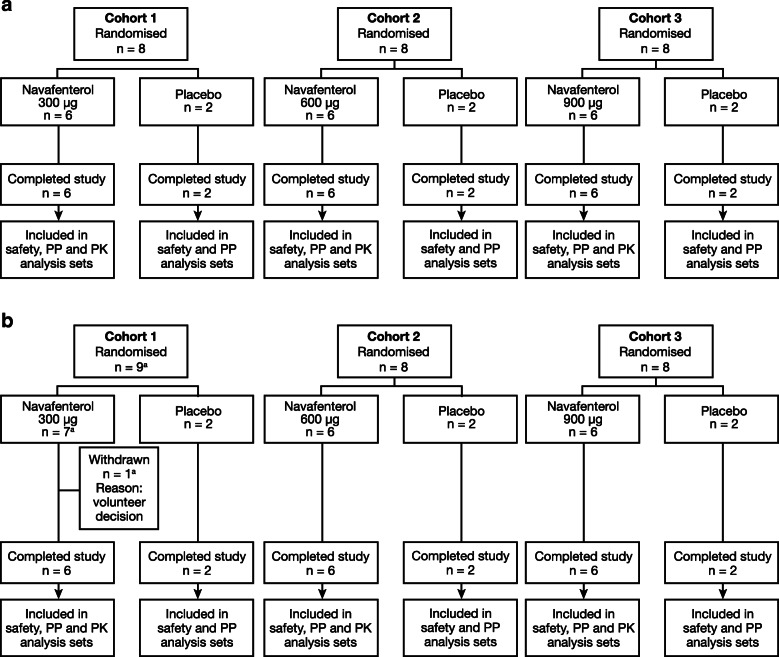
Patient disposition and flow in (**a**) study A and (**b**) study B. PK = pharmacokinetic; PP = per protocol. ^a^One volunteer withdrew prior to the first dose and was replaced by a reserve volunteer as per the protocol

Twenty-four volunteers completed each study (8 volunteers per cohort: 6 received navafenterol, 2 received placebo; Fig. 2). In study B, 1 volunteer (navafenterol 300 μg) withdrew prior to the first dose and was replaced by a reserve volunteer as per the protocol. There were no other discontinuations. Demographics and baseline characteristics were generally similar between treatment groups in each study and, with the exception of race and ethnicity, were similar between the studies (Table 1).

**Table 1:** Patient Demographics and Baseline Characteristics in Studies A and B (Safety Population)

	Study A				Study B			
	Placebo *n* = 6	Navafenterol 300 μg *n* = 6	Navafenterol 600 μg *n* = 6	Navafenterol 900 μg *n* = 6	Placebo *n* = 6	Navafenterol 300 μg *n* = 6	Navafenterol 600 μg *n* = 6	Navafenterol 900 μg *n* = 6
Age, years	41.5 (7.6)	34.0 (5.7)	44.2 (7.9)	36.5 (6.8)	35.7 (6.4)	30.0 (5.0)	32.0 (7.8)	35.5 (8.5)
Race, n (%)								
Asian	0	1 (16.7)	0	0	6 (100.0)	6 (100.0)	6 (100.0)	6 (100.0)
Black/African American	0	0	0	1 (16.7)	0	0	0	0
White	6 (100.0)	5 (83.3)	6 (100.0)	5 (83.3)	0	0	0	0
Ethnicity, n (%)								
Hispanic or Latino	1 (16.7)	0	1 (16.7)	0	0	0	0	0
Height, cm	179.7 (4.7)	179.7 (6.3)	174.2 (8.0)	176.8 (3.3)	168.5 (4.7)	172.0 (2.6)	172.8 (5.6)	170.8 (6.3)
Weight, kg	78.0 (11.1)	85.2 (13.2)	75.8 (5.25)	76.2 (8.4)	64.3 (5.1)	62.8 (5.3)	68.0 (9.3)	64.8 (6.2)
Body mass index, kg/m^2^	24.1 (2.6)	26.2 (2.3)	24.8 (3.7)	24.3 (1.8)	22.7 (2.0)	21.2 (1.3)	22.7 (2.1)	22.2 (2.4)

Data are mean (standard deviation) unless otherwise specified

### Safety

#### Adverse events

There were no deaths, serious AEs or treatment-emergent AEs (AEs with onset after the first dose [TEAEs]) leading to discontinuation of navafenterol in either study. TEAEs occurring in ≥2 volunteers in any treatment group in either study are presented in Table 2; all TEAEs were of mild severity in the placebo group and of mild-to-moderate severity in the navafenterol groups. The most frequent TEAEs overall were vessel puncture-site bruise (placebo, *n* = 2; navafenterol 900 μg, *n* = 3) in study A and diarrhoea (placebo, *n* = 1; navafenterol 300 μg, n = 2; navafenterol 900 μg, n = 3) in study B. TEAEs considered related to treatment, as assessed by the site investigator, were headache (navafenterol 300 μg, n = 2), oropharyngeal pain (placebo, n = 1) and cough (placebo, n = 1) in study A and headache (navafenterol 900 μg, n = 1), diarrhoea (navafenterol 300 μg, n = 2), chest discomfort (navafenterol 300 μg, n = 1), thirst (navafenterol 300 μg, n = 1), increased hepatic enzymes (navafenterol 600 μg, n = 1) and somnolescence (placebo, n = 1) in study B. No dose-response relationship was observed for any TEAEs.

**Table 2:** Frequency and Intensity of TEAEs Overall and TEAEs Occurring in ≥2 Volunteers in Any Treatment Group in Either Study A or B, by MedDRA^a^ Preferred Term (Safety Population)

	Study A				Study B			
	Placebo *n* = 6	Navafenterol 300 μg *n* = 6	Navafenterol 600 μg *n* = 6	Navafenterol 900 μg *n*n = 6	Placebo *n* = 6	Navafenterol 300 μg *n* = 6	Navafenterol 600 μg *n* = 6	Navafenterol 900 μg *n* = 6
Any TEAE, n (%)	4 (66.7)	3(50.0)	1 (16.7)	5 (83.3)	3 (50.0)	4 (66.7)	5 (83.3)	5 (83.3)
Mild	4 (66.7)	3 (50.0)	1 (16.7)	5 (83.3)	3 (50.0)	4 (66.7)	4 (66.7)	4 (66.7)
Moderate	0	2 (33.3)	1 (16.7)	0	0	0	1 (16.7)	1 (16.7)
Diarrhea	0	0	0	0	1 (16.7)	2 (33.3)	0	3 (50.0)
Mild	0	0	0	0	1 (16.7)	2 (33.3)	0	3 (50.0)
Vessel puncture site bruise^b^	2 (33.3)	0	0	3 (50.0)	0	0	2 (33.3)	0
Mild	2 (33.3)	0	0	3 (50.0)	0	0	2 (33.3)	0
Headache	0	2 (33.3)	1 (16.7)	0	0	0	0	1 (16.7)
Mild	0	1 (16.7)	0	0	0	0	0	1 (16.7)
Moderate	0	1 (16.7)	1 (16.7)	0	0	0	0	0
Dermatitis contact	0	0	0	1 (16.7)	0	0	2 (33.3)	0
Mild	0	0	0	1 (16.7)	0	0	2 (33.3)	0
Rash	0	0	0	0	1 (16.7)	0	0	2 (33.3)
Mild	0	0	0	0	1 (16.7)	0	0	2 (33.3)
Nasopharyngitis	0	0	0	2 (33.3)	0	0	0	0
Mild	0	0	0	2 (33.3)	0	0	0	0
Oropharyngeal pain	2 (33.3)	0	0	0	0	0	0	0
Mild	2 (33.3)	0	0	0	0	0	0	0

^a^MedDRA version 19.0

^b^‘Catheter site bruise’ in study B

*MedDRA* Medical Dictionary for Regulatory Activities, *n* number of patients, *TEAE* treatment emergent adverse event

#### Clinical laboratory assessments

**Fig. 3:**
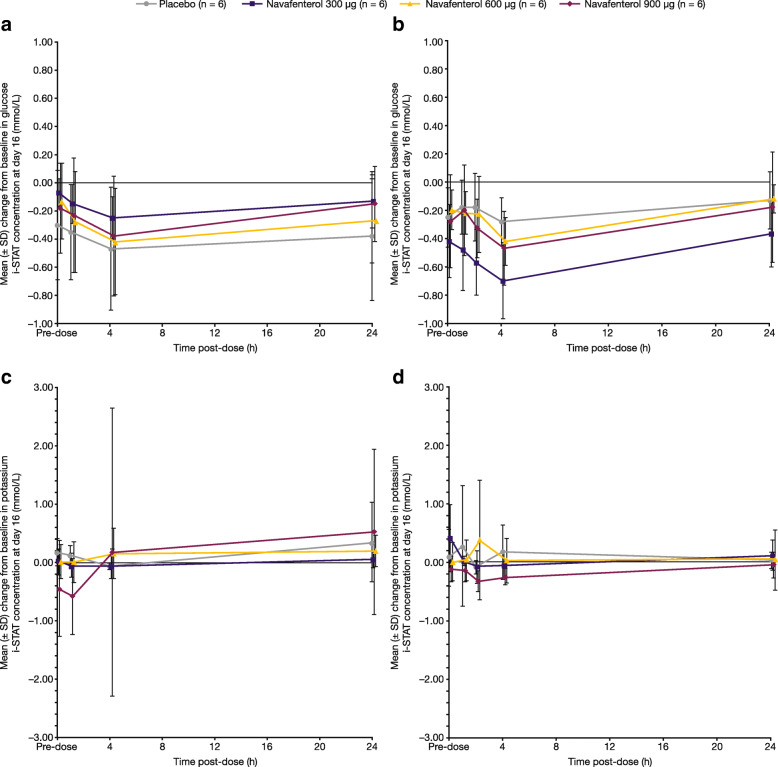
Mean change from baseline on day 16 (safety population) in glucose i-STAT concentration in (**a**) study A and (**b**) study B, and mean change from baseline in potassium i-STAT concentration in (**c**) study A and (**d**) study B. SD = standard deviation

Overall, there were no clinically significant haematology, clinical chemistry or urinalysis findings in either study. One volunteer had alanine aminotransferase values above the upper limit of normal (navafenterol 600 μg; study B); this did not meet the criteria of Hy’s Law and was reported as a TEAE. There were no clinically relevant trends in serum glucose or potassium concentrations over time; results on day 16 are presented in Fig. 3.

#### ECG, telemetry and vital signs

**Fig. 4:**
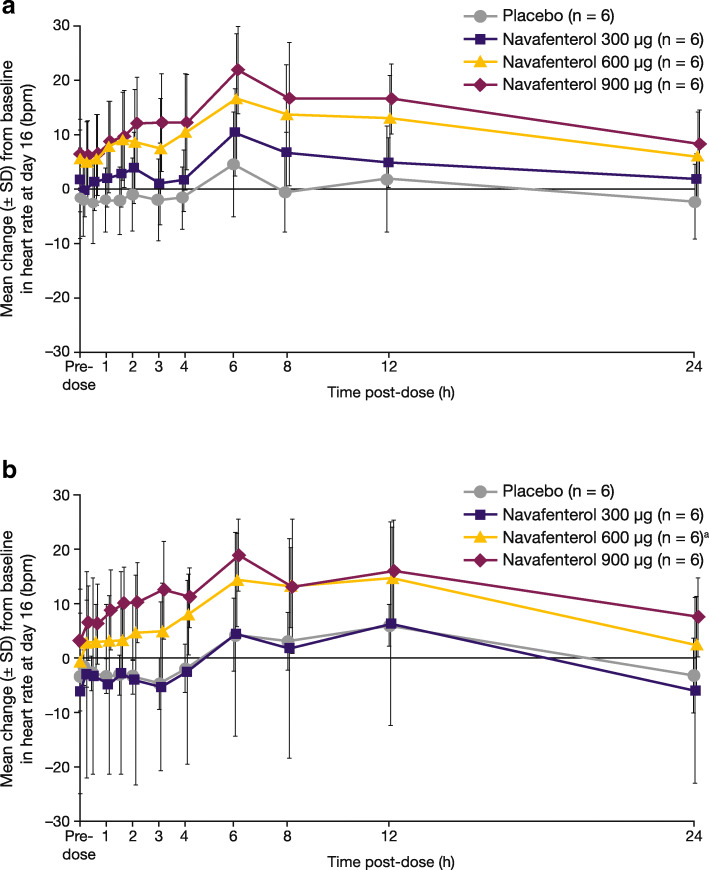
Mean change from baseline in heart rate over time at day 16 (safety population) in (**a**) study A and (**b**) study B. ^a^except for navafenterol 600 μg at 12 h post dose on day 16, *n* = 5. bpm = beats per minute; SD = standard deviation

No trends or dose response were observed in mean change from baseline in Fridericia’s corrected QT interval (QTcF) after multiple dosing and no QTcF outlier values were observed. There were no clinically significant changes in other ECGs or telemetry and no significant vital sign abnormalities. Based on vital signs data, collected at pre-dose on all dosing days and also at 1, 2, 4, 12 and 24 h post dose on Days 1, 5, 7, 10, 12 and 16, an increase of heart rate was observed over time, with a trend towards stabilisation after 7–10 days of repeated administration of navafenterol, after PK steady state was achieved. Based on digital ECGs, a dose dependent increase was registered in heart rate at day 16 in both studies (Fig. 4).

#### Physical examination

Physical examination findings in both studies, reported as TEAEs, occurring in ≥2 volunteers in any treatment arm were vessel puncture/catheter site bruise and contact dermatitis (Table 2).

### Pharmacokinetics

**Fig. 5:**
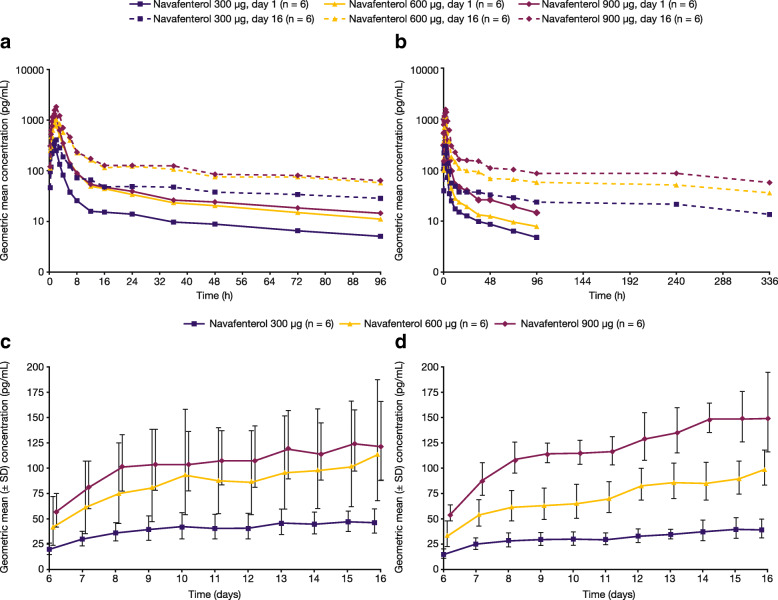
Geometric mean plasma concentration-time profiles of navafenterol (pharmacokinetic population) on days 1 and 16 in (**a**) study A and (**b**) study B, and pre-dose concentrations from days 6 to 16 in (**c**) study A and (**d**) study B. Note that a longer sampling scheme was used in study B (samples collected up to 336 h following the last dose) than in study A (samples collected up to 96 h following the last dose). SD = geometric standard deviation

Both studies showed rapid absorption of navafenterol on day 1 (median t_max_ range: 1.01–1.52 h; Table 3; Fig. 5a and b), with similar results after multiple dosing (Table 4). Steady state for navafenterol was achieved after approximately 8 and 10 days of dosing in study A (Fig. 5c) and study B (Fig. 5d), respectively. At steady state, after t_max_ was achieved, plasma concentrations declined in a biexponential manner, with mean t_½λz_ of 62.4–70.2 h in non-Japanese volunteers and 209.2–250.7 h in Japanese volunteers on day 16. Increases in exposure (C_max_ and AUC) with increasing dose were generally dose proportional, although there was some statistical evidence of greater than dose-proportional increases in exposure of navafenterol after multiple dosing in study B, with the 95% confidence intervals for the slope estimates excluding unity on day 16 (e-Table 1). No significant time dependency was observed for navafenterol in either study (e-Table 2). Both studies showed some evidence of accumulation of navafenterol with multiple dosing (Rac [C_max_] range 0.90–2.37; Rac [AUC_0–24_] range 1.39–3.06; Table 4).

**Table 3:** Plasma PK Parameters for Navafenterol on Day 1 (PK Population)

	Study A			Study B		
	Navafenterol 300 μg	Navafenterol 600 μg	Navafenterol 900 μg	Navafenterol 300 μg	Navafenterol 600 μg	Navafenterol 900 μg
AUC_0–24_, pg.h/mL						
n	6	5	6	6	6	6
Geometric mean	1272	3805	4862	1208	2066	4460
%GCV	18.69	58.72	15.58	22.89	61.19	18.69
AUC_last_, pg.h/mL						
n	6	5	6	6	6	6
Geometric mean	1864	5161	6464	1770	2886	6134
%GCV	19.20	63.64	16.37	18.83	61.28	21.39
C_max_, pg/mL						
n	6	5	6	6	6	6
Geometric mean	397.0	991.1	1568	352.4	580.4	1151
%GCV	28.66	63.45	20.41	28.52	63.64	31.74
t_max_, h						
n	6	5	6	6	6	6
Median	1.50	1.52	1.51	1.01	1.25	1.50
Min–max	1.00–1.52	1.50–1.55	1.48–1.52	1.00–1.48	0.75–1.50	1.00–2.00
t_½λz_, h						
n	6	5	6	6	6	6
Arithmetic mean	50.44	42.89	49.24	52.44	51.75	50.51
SD	6.796	11.99	9.055	8.207	7.586	6.213

*AUC* area under the curve, *AUC*_*0–24*_ AUC from 0 to 24 h, *AUC*_*last*_ AUC from time 0 to time of the last quantifiable measurable concentration, *C*_*max*_ maximum plasma concentration, *%GCV* geometric coefficient of variation, *max* maximum, *min* minimum, *n* number of non-missing observations, *PK* pharmacokinetic, *SD* standard deviation, *t*_*½λz*_ terminal half-life, *t*_*max*_ time to reach Cmax

**Table 4:** Steady-State Plasma PK Parameters for navafenterol on Day 16 (PK Population)

	Study A			Study B^a^		
	Navafenterol 300 μg	Navafenterol 600 μg	Navafenterol 900 μg	Navafenterol 300 μg	Navafenterol 600 μg	Navafenterol 900 μg
AUC_0–24_, pg.h/mL						
n	6	5	6	6	6	6
Geometric mean	2103	5508	7077	1633	5596	8332
%GCV	25.20	47.25	19.87	42.47	23.31	16.78
AUC_last_, pg.h/mL						
n	6	6	6	6	6	6
Geometric mean	4432	10,380	12,570	7167	19,340	30,470
%GCV	26.17	52.30	21.75	30.23	19.05	22.20
C_max_, pg/mL						
n	6	6	6	6	6	6
Geometric mean	406.5	1018	1830	297.2	1220	1559
%GCV	34.44	53.64	28.47	69.43	35.03	28.02
t_max_, h						
n	6	6	6	6	6	6
Median	1.50	1.50	1.50	1.04	1.49	1.50
Min–max	1.50–1.53	1.48–1.50	1.50–1.50	0.75–1.50	0.98–2.00	1.00–1.98
t_½λz_, h						
n	6	5	5	6	6	6
Arithmetic mean	78.51	62.39	70.19	209.2	236.0	250.7^a^
SD	11.01	16.69	4.144	40.76	34.54	67.04
Rac (C_max_)						
n	6	5	6	6	6	6
Arithmetic mean	1.032	1.054	1.181	0.9047	2.372	1.362
Min–max	0.881–1.23	0.979–1.12	0.843–1.40	0.391–1.30	1.26–5.10	1.16–1.55
Rac (AUC_0–24_)						
n	6	5	6	6	6	6
Arithmetic mean	1.675	1.451	1.463	1.393	3.062	1.879
Min–max	1.33–2.14	1.25–1.60	1.20–1.58	0.891–1.88	1.71–6.87	1.54–2.09

^a^Note that a longer sampling scheme was used in study B (samples collected up to 336 h following the last dose) compared with study A (samples collected up to 96 h following the last dose)

*AUC* area under the curve, *AUC*_*0–24*_ AUC from 0 to 24 h, *AUC*_*last*_ AUC from time 0 to time of the last quantifiable measurable concentration, *C*_*max*_ maximum plasma concentration, *%GCV* geometric coefficient of variation, *max* maximum, *min* minimum, *n* number of non-missing observations, *PK* pharmacokinetic, *Rac (C*_*max*_*)* accumulation ratio for C_max_, *Rac (AUC*_*0–24*_*)* accumulation ratio for AUC_0–24_, *SD* standard deviation, *t*_*½λz*_ terminal half-life, *t*_*max*_ time to reach C_max_

The metabolite to parent ratio on day 16 was similar between studies for LAS191861 (MRC_max_: study A, ~ 11–14%; study B, ~ 9–13%; MRAUC_0–24_: study A, ~ 23–28%; study B, ~ 19–29%) and LAS34850 (MRC_max_: study A, ~ 136–230%; study B, ~ 145–373%; MRAUC_0–24_: study A, ~ 396–431%; study B, ~ 282–550%).

Pharmacodynamic results are provided in e-Appendix 2.

## Discussion

Overall, these first safety, tolerability and PK studies of the dual pharmacology MABA, navafenterol, in healthy volunteers identified no safety concerns. There was no dose-response relationship in the pattern of TEAEs, clinical laboratory assessments (including serum glucose and potassium) or ECG results (including QTc interval) at the collective level or in individual volunteers, with the exception of a dose-dependent response effect on heart rate at steady state (day 16). In both studies, dosing remained below prespecified human exposure limits and the maximum tolerated dose was not reached. These two studies were performed in healthy volunteers and were not, therefore, designed to examine the efficacy of navafenterol. No notable trends or abnormalities were observed in exploratory lung function parameters in either study.

The increase in heart rate observed following multiple dosing with navafenterol in both studies is one of the potential effects expected for navafenterol in healthy volunteers naïve to LABA bronchodilators, based on the β_2_-adrenoceptor agonist component of its dual bronchodilator effect [10]. This effect was not noted in a 14-day study of patients (*n* = 42) with COPD treated with navafenterol and there were no other significant changes in vital signs, ECG results, or clinical laboratory tests in this population [11]. However, given the small number of participants in studies A and B, this effect does require further monitoring in future studies.

Race and ethnicity can affect drug exposure and response. A review of new drugs approved by the FDA between 2008 and 2013 found that one-fifth demonstrated differences in exposure and/or response across racial/ethnic groups [9]. In the current studies, there was no indication of a difference in exposure of navafenterol between healthy male non-Japanese and Japanese volunteers. Steady state for navafenterol was achieved after 8 days of multiple dosing in non-Japanese volunteers and after 10 days of multiple dosing in Japanese volunteers. Due to a longer than anticipated t_½λz_ of navafenterol, the t_½λz_ values were generally calculated over a period of less than the desired three half-lives and this may have led to unreliable estimates. The substantially longer t_½λz_ observed for navafenterol in Japanese volunteers compared with non-Japanese volunteers was the result of a longer PK sampling period following the last dose in study B (336 h) compared with study A (96 h). Based on the time to achieve steady state and the accumulation, the ‘effective’ half-life was substantially shorter, with approximate values of 48 h in both studies. The t_½λz_ was consistent across the dose range in both studies. The PK of navafenterol appeared to be linear with dose and time*.*

## Conclusion

Multiple ascending doses of navafenterol (300, 600 and 900 μg) were well-tolerated in healthy male volunteers. No safety concerns were identified and stopping criteria were not met in either study. The safety and PK of navafenterol were similar in non-Japanese and Japanese volunteers. The findings of these studies support further clinical development of navafenterol.

## Supplementary information

**Additional file 1 : e-Appendix 1**. Methods. **e-Appendix 2**. Results. **E-Table** 1 Assessment of Dose Proportionality of Navafenterol in Studies A and B. **E-Table** 2 Assessment of Time-Dependency of Navafenterol in Studies A and B.

## Data Availability

Data underlying the findings described in this manuscript may be obtained in accordance with AstraZeneca’s data sharing policy described at https://astrazenecagrouptrials.pharmacm.com/ST/Submission/Disclosure.

## Abbreviations

AE: Adverse event
AUC: Area under the concentration-time curve from time zero extrapolated to infinity
AUC_0–24_: Area under the concentration-time curve from time zero to 24 h post-dose
AUC_last_: Area under the concentration-time curve from time zero to the time of last quantifiable concentration
C_max_: Maximum concentration
COPD: Chronic obstructive pulmonary disease
ECG: Electrocardiogram
GOLD: Global Initiative for chronic obstructive lung disease
LABA: Long-acting β_2_ adrenoceptor agonist
LAMA: Long-acting muscarinic receptor antagonist
MABA: Muscarinic receptor antagonist and β_2_ adrenoceptor agonist
MRAUC_0–24_: Metabolite to parent ratio for AUC_0–24_
MRC_max_: Metabolite to parent ratio for C_max_
PK: Pharmacokinetic
QTcF: Fridericia’s corrected QT interval
Rac (AUC_0–24_): Accumulation ratio for AUC_0–24_
Rac (C_max_): Accumulation ratio for C_max_
t_½λz_: Terminal half-life
TEAE: Treatment-emergent adverse event
t_max_: Time to reach maximum concentration

## Footnotes

1 Registered trademarks of the AstraZeneca group of companies; for use within the USA as Pressair® and Genuair™ within all other licensed territories
