# Supplementary material for: Navafenterol (AZD8871) in healthy volunteers: safety, tolerability and pharmacokinetics of multiple ascending doses of this novel inhaled, long-acting, dual-pharmacology bronchodilator, in two phase I, randomised, single-blind, placebo-controlled studies
Source: Respir Res. 2020 Sep 9;21(Suppl 1):212. doi: 10.1186/s12931-020-01474-1 (PMC7488005; doi:10.1186/s12931-020-01474-1)
Supplement: Supplementary file 1 — Additional file 1 : e-Appendix 1. Methods. e-Appendix 2. Results. E-Table 1 Assessment of Dose Proportionality of Navafenterol in Studies A and B. E-Table 2 Assessment of Time-Dependency of Navafenterol in Studies A and B. [file 12931_2020_1474_MOESM1_ESM.docx]

**Online Supplement**

# e-Appendix 1. Methods

## Study Design

**Study dates:** Study A was conducted between June 22 and November 28, 2016 and study B between June 19 and October 13, 2017.

**Ethics committees:** Study A was approved by the London – Brent Research Ethics Committee, London, UK, (Reference No: 16/LO/0456) and study B was approved by the Research Ethics Committee South Central - Berkshire B Health Research Authority, Bristol, UK (Reference No: 17/SC/0127).

**Randomisation:** Randomisation was performed on the morning of the first administration of study treatment. Randomisation codes were assigned strictly sequentially. Within each cohort, volunteers were randomised to navafenterol (AZD8871) or placebo in a ratio of 6:2 (active: placebo). The randomisation list was provided to the pharmacist, the personnel responsible for study medication preparation, the personnel analysing the pharmacokinetic (PK) samples and the investigator before the experimental phase of the trial to ensure correct study medication administration.

**Blinding:** Both studies were single blind, with only the volunteers participating in the study not knowing what treatment they were receiving. The investigator and sponsor knew which treatment each patient received; however, in study B this information was only revealed to the investigator/sponsor at the time of the Safety Review Committee meetings. Placebo-containing dry-powder inhaler devices presented with the same external appearance as the navafenterol devices, and had the same composition except for the active ingredient.

**Stopping criteria:** If any of the following scenarios occurred within a cohort with a reasonable possibility of a causal relationship with navafenterol, dose escalation was to be stopped:

- **≥**1 volunteer who received navafenterol reported a serious adverse event (AE) or experienced AEs judged to be non-tolerable and causally related to navafenterol
- **≥**2 volunteers experienced severe/clinically significant AEs at least possibly related to the study drug
- **≥**2 volunteers, who received navafenterol developed any of the following:
  - QTc prolongation, defined as Fridericia’s corrected QT interval (QTcF) > 500 ms, or a prolongation from baseline of > 60 ms, confirmed (persistent for at least 5 min) and determined post-dose either during continuous 12-lead electrocardiogram (ECG) monitoring or on a repeat 12-lead ECG
  - symptomatic bradycardia, defined as resting supine heart rate < 40 beats per minute (bpm) or asymptomatic bradycardia defined as resting supine heart rate < 30 bpm while awake, persisting for at least 10 min.
  - hypertension, defined as an increase in resting supine systolic blood pressure > 40 mmHg to above 180 mmHg, persisting for at least 10 min
  - alanine aminotransferase (ALT) or aspartate aminotransferase (AST) > 3× the upper limit of normal (ULN) or bilirubin or alkaline phosphatase > 2× ULN
  - confirmed (two subsequent laboratory measurements) leukocyte count < 2.0 × 10^9^/L, neutrophil count < 1.0 × 10^9^/L , platelet count < 75 × 10^9^/L, serum potassium levels < 2.8 mmol/L, or serum creatinine increase to > 1.5× ULN
  - hypotension, defined as an asymptomatic fall in systolic blood pressure > 20 mmHg to below 70 mmHg persisting for at least 10 min, or a symptomatic fall in resting supine systolic blood pressure > 20 mmHg (excluding vasovagal reaction)
  - tachycardia, defined as resting supine heart rate > 125 bpm, persisting for at least 10 min
  - other clinically significant changes in laboratory values or safety parameters
- ≥ 2 volunteers who received navafenterol in a cohort, or ≥ 3 volunteers in total who received navafenterol, had a reduction in forced expiratory volume in 1 second (FEV_1)_ ≥ 30% of the pre-dose value within 1 h after administration of the study drug
- ≥ 1 volunteer who received navafenterol fulfilled Hy’s Law, defined as an increase in AST or ALT ≥ 3× ULN and total bilirubin > 2× ULN, where no other reason could be found to explain the combination of increases

PK criteria for halting dose escalation were achievement or predicted achievement of the maximum steady state exposure level (observed maximum concentration [C_max_] 3500 pg/mL and/or area under the concentration-time curve from time zero to 24 h post-dose [AUC_0‑24_] 14,000 pg.h/mL).

## Volunteers

Additional inclusion criteria included: body mass index ≥ 18 and ≤ 30 kg/m^2^ and weight ≥ 50 kg, FEV_1_ and forced vital capacity (FVC) ≥ 80% predicted value,^1^ systolic blood pressure ≥ 90 and ≤ 140 mmHg, diastolic blood pressure ≥ 50 and ≤ 90 mmHg and heart rate ≥ 45 and ≤ 90 bpm in resting supine position, and negative hepatitis B surface antigen, hepatitis B core antibody (immunoglobulin M), hepatitis C antibody and human immunodeficiency virus I and II antibodies.

Exclusion criteria included clinically significant illness or medical/surgical procedure 4 weeks prior to screening, history of malignancy within the past 5 years (with the exception of localised basal cell carcinoma of the skin), current smoker, smoking history during the past 6 months or smoking history of > 10 pack-years, current use or use within the past 6 months of electronic cigarettes or other forms of nicotine, prolonged QT corrected using QTcF interval > 450 ms, family history of long QT syndrome, clinically significant abnormalities in rhythm, conduction or morphology noted on physical examination, ECG or telemetry recording, history of alcohol or drug abuse in the past 2 years, donation or loss of > 400 mL blood and plasma within the previous 3 months and any laboratory abnormality or suspicion of clinically relevant disease, which, in the opinion of the investigator, could put the volunteer at risk.

Consumption of some items was restricted during the studies: alcohol or energy drinks containing taurine or glucuronolactone from 72 h prior to admission to final follow-up; caffeine-containing drinks for 24 h prior to admission to the unit until discharge from the unit (excessive caffeine intake was also to be avoided between study visits); poppy seeds from consent to final follow-up; grapefruit or Seville orange products from 7 days prior to admission to final follow-up. Strenuous physical activity outside of the patient’s usual routine was not allowed from 72 h prior to screening until the final follow-up visit.

Volunteers were required to fast for at least 8 h overnight before morning administration of the study drug. A moderate amount of water (up to 500 mL) was allowed up to 1 h before administration and could be resumed 1 h post-administration. A meal was provided 4 h after dosing on days 1, 5, 10 and 16, and 1 h after dosing on all other days.

## Assessments

**Safety**: AEs were recorded throughout the study. Physical examinations were performed at screening, day −1, days 1–20 and at final follow-up. Vital signs were recorded at screening, day −1 and pre-dose on all dosing days, and also at 1, 2, 4, 12 and 24 h post-dose on days 1, 5, 7, 10, 12 and 16 and at final follow-up. Clinical laboratory assessments were performed at baseline, on day 20 prior to discharge, and at final follow-up. Additionally, in study A assessments were performed on day 1 at 24 h post-dose, day 10 at pre-dose, and day 16 at pre-dose and 24 h post-dose. Volunteers fasted for 8 h prior to clinical laboratory testing. i-STAT (potassium and glucose) measurements were performed pre-dose and at 1 and 2 h (study B only), 4 and 24 h post-dose on days 1, 5, 10 and 16 and at final follow-up. Volunteers fasted for 4 h prior to i-STAT recordings. 12-lead digital ECG (dECG) was recorded at 0.25, 0.5, 1, 1.5, 2, 3, 4, 6, 8, 12, 24, 48 and 72 h following the first (day 1) and last (day 16) dose. Safety ECG recordings were performed at the end of each dECG recording. 2-lead real-time telemetry was performed on day −1 for at least 4 h and on days 1, 10 and 16 from 30 min pre-dose to 24 h post-dose.

**Pharmacodynamics**: Spirometry was performed at screening and within 90 min pre-dose and at 80 min and 5 and 23 h post-dose on days 1, 5 and 16 and within 90 min pre-dose on day 10. FEV_1_ and FVC assessments were performed according to the acceptability and reproducibility criteria set out by the American Thoracic Society/ European Respiratory Society recommendations. Pupillometry was performed in study A only, on day −1 (familiarisation test), day 1 at pre-dose and 1.5 h post-dose and day 16 pre-dose and 1.5 and 23 h post-dose, using a Procyon P3000 USB Pupillometer (Clement Clarke International/ Haag-Streit UK Ltd, Harlow, UK). The pupil size of both eyes was measured under three different conditions: 0.04 lux (scotopic), 0.4 lux (low mesopic) and 4.0 lux (high mesopic).

## Statistical Analysis

All statistical analyses were performed using SAS^®^ Version 9.3 software (SAS Institute, Cary, NC) or later.

**Pharmacokinetics***:* PK parameters were derived using Phoenix™ WinNonlin^®^ Version 6.4 for non-compartmental analysis.

The AUC was derived using trapezoidal methods when concentrations were increasing and the logarithmic trapezoidal method when concentrations were decreasing. For derivation of λ_z_ and t_½λz_, the R_sq_adj_ (goodness of fit) value needed to be greater than 0.8 for at least three measured concentrations included in the regression. In those cases where % extrapolated AUC was higher than 20%, λ_z_ and derived parameters were excluded from the summary statistics.

Dose proportionality of navafenterol after single dose (day 1) and multiple dose (day 16) were analysed using the power model approach with the natural logarithm of the PK parameter (AUC and C_max_ [day 1] and AUC_0–24_ and C_max_ [day 16]) as the dependent variables and the natural logarithm of the dose as the independent variable:

log (dependent variable)=α + β•log(dose).

The intercept α and the slope β together with 2-sided 90% confidence interval were estimated. If AUC was not robustly estimated, AUC_last_ on day 1 could be used for dose-proportionality assessment instead of AUC.

Time dependency was evaluated by comparing AUC_0–24_ (day 16) with AUC (day 1) and accumulation was evaluated by comparing AUC_0–24_ (day 16) with AUC_0–24_ (day 1) and C_max_ (day 16) with C_max_ (day 1). A linear mixed-effect model was used with the logarithm of the above PK parameters as the response variable, and dose, day and dose by day interaction as fixed effects. Day was treated as a repeated effect within volunteer.

**Pharmacodynamics**: Lung function data were analysed in the per-protocol population, which consisted of all volunteers who received at least one dose of navafenterol or placebo and who had at least one pre- and post-dose measurement for FEV_1_ with no relevant protocol deviations. To explore the change from baseline for trough and peak FEV_1_ for each navafenterol dose compared with pooled placebo an analysis of covariance model was used with treatment as fixed effect and the baseline value (pre-dose day 1) as a covariate. Separate tests were performed for each parameter and timepoint. If the model did not converge, separate statistical tests for each navafenterol dose vs pooled placebo were performed.

Pupillometry data were analysed in the safety population. Mean and peak pupil diameter were generated from PupilFit software for each eye under each measurement condition.

## Reference

1. Quanjer PH Stanojevic S, Cole TJ et al. Multi-ethnic reference values for spirometry for the 3–95-yr age range: the global lung function 2012 equations. *Eur Respir J.* 2012;40(6):1324-1343.

# e-Appendix 2. Results

## Pharmacodynamics

Changes from baseline in trough and peak FEV_1_ were greater with navafenterol than with placebo, but there was no notable trend in the changes from baseline with increasing dose of navafenterol in either study. In study A, there were no statistically significant differences in change from baseline in trough FEV_1_ vs placebo at any of the time points analysed, but in study B there was a significant increase in trough FEV_1_ vs placebo for the 600 and 900 µg dose on day 6 (least squares [LS] mean difference 268 and 209 mL, respectively, both *P* < .05), and day 17 (LS mean difference 256 and 193 mL, respectively, *P* < .05) and the 600 µg dose on day 10 (232 mL, *P* < .01). In both studies, change from baseline in peak FEV_1_ was significantly increased vs placebo for all doses of navafenterol on day 1 (LS mean difference range: study A, 207–379; study B, 156–225 mL; all *P* < .05). On day 5, a significant increase in change from baseline in peak FEV_1_ was only seen in study B with navafenterol 600 µg (LS mean difference 274 mL, *P* < .05). On day 16, change from baseline in peak FEV_1_ was significantly increased vs placebo with navafenterol 300 µg and navafenterol 600 µg in study A (LS mean difference 265 and 226 mL, respectively, both *P* < .05) and navafenterol 600 µg and navafenterol900 µg in study B (LS mean difference 239 and 190 mL, respectively, both *P* < .05) on day 16.

Pupillometry results followed expected trends following increasing illumination intensity

(decreased pupil diameter with increased illumination intensity). There were no clinically relevant changes from baseline in mean pupil diameter with navafenterol treatment.

**e-Table 1** Assessment of Dose Proportionality of Navafenterol in Studies A and B

|  | Study A | | | |  | Study B | | | |  |
| --- | --- | --- | --- | --- | --- | --- | --- | --- | --- | --- |
| Parameter | n | Slope estimate | SE | 95% CI |  | n | Slope estimate | SE | 95% CI | |
| Day 1 |  |  |  |  |  |  |  |  |  | |
| C_max_ (pg/mL) | 17 | 1.257 | 0.188 | 0.856, 1.658 |  | 18 | 1.039 | 0.218 | 0.578, 1.501 | |
| AUC_last_ (h.pg/mL) | 17 | 1.173 | 0.184 | 0.782, 1.565 |  | 18 | 1.085 | 0.198 | 0.665, 1.506 | |
| AUC (h.pg/mL) | 16 | 1.127 | 0.190 | 0.719, 1.535 |  | 16 | 1.080 | 0.227 | 0.594, 1.566 | |
| Day 16 |  |  |  |  |  |  |  |  |  | |
| C_max_ (pg/mL) | 18 | 1.365 | 0.194 | 0.955, 1.775 |  | 18 | 1.565 | 0.241 | 1.054, 2.077 | |
| AUC_0–24_ (h.pg/mL) | 18 | 1.135 | 0.167 | 0.780, 1.490 |  | 18 | 1.515 | 0.153 | 1.190, 1.840 | |

AUC = area under the curve; AUC_0–24_ = AUC from 0 to 24 h; AUC_last_ = AUC from time 0 to time of the last quantifiable measurable; CI = confidence interval; C_max_ = maximum concentration; n = number of data points included in the analysis.

**e-Table 2** Assessment of Time-Dependency of Navafenterol in Studies A and B

|  | | Study A | | |  | Study B | | |
| --- | --- | --- | --- | --- | --- | --- | --- | --- |
| Dose of navafenterol | Parameter | n | Ratio Day 16/Day 1 | 95% CI |  | n | Ratio Day 16/Day 1 | 95% CI |
| 300 µg |  |  |  |  |  |  |  |  |
| Day 1 | AUC (h.pg/mL) | 6 | 0.949 | 0.675, 1.334 |  | 5 | 0.729 | 0.506, 1.049 |
| Day 16 | AUC_0–24_ (h.pg/mL) | 6 |  |  |  | 5 |  |  |
| 600 µg |  |  |  |  |  |  |  |  |
| Day 1 | AUC (h.pg/mL) | 4 | 0.980 | 0.230, 4.167 |  | 5 | 1.641 | 0.799, 3.370 |
| Day 16 | AUC0–24 (h.pg/mL) | 4 |  |  |  | 5 |  |  |
| 900 µg |  |  |  |  |  |  |  |  |
| Day 1 | AUC (h.pg/mL) | 6 | 0.944 | 0.732, 1.218 |  | 6 | 1.156 | 0.981, 1.362 |
| Day 16 | AUC_0–24_ (h.pg/mL) | 6 |  |  |  | 6 |  |  |

AUC = area under the curve; AUC_0–24_ = AUC from 0 to 24 h; CI = confidence interval; n = number of data points included in the analysis.
